# Traditional Approaches and Emerging Biotechnologies in Grapevine Virology

**DOI:** 10.3390/v15040826

**Published:** 2023-03-24

**Authors:** Giulia Tarquini, Mattia Dall’Ara, Paolo Ermacora, Claudio Ratti

**Affiliations:** 1 Department of Agricultural, Environmental, Food and Animal Sciences (Di4A), University of Udine, 33100 Udine, Italy; paolo.ermacora@uniud.it; 2 Department of Agricultural and Food Sciences (DISTAL), University of Bologna, 40127 Bologna, Italy; mattia.dallara5@unibo.it (M.D.); claudio.ratti@unibo.it (C.R.)

**Keywords:** vitis vinifera, transgenesis, virus resistance, genome editing, RNAi, vectors

## Abstract

Environmental changes and global warming may promote the emergence of unknown viruses, whose spread is favored by the trade in plant products. Viruses represent a major threat to viticulture and the wine industry. Their management is challenging and mostly relies on prophylactic measures that are intended to prevent the introduction of viruses into vineyards. Besides the use of virus-free planting material, the employment of agrochemicals is a major strategy to prevent the spread of insect vectors in vineyards. According to the goal of the European Green Deal, a 50% decrease in the use of agrochemicals is expected before 2030. Thus, the development of alternative strategies that allow the sustainable control of viral diseases in vineyards is strongly needed. Here, we present a set of innovative biotechnological tools that have been developed to induce virus resistance in plants. From transgenesis to the still-debated genome editing technologies and RNAi-based strategies, this review discusses numerous illustrative studies that highlight the effectiveness of these promising tools for the management of viral infections in grapevine. Finally, the development of viral vectors from grapevine viruses is described, revealing their positive and unconventional roles, from targets to tools, in emerging biotechnologies.

## 1. General Perspectives on Viral Diseases in Grapevine

Grapevine (*Vitis vinifera*) is one of the world’s most significant fruit crops, standing out among cultivated plant species for its paramount socio-economic relevance. According to 2020 statistics from the Food and Agriculture Organization (FAO) of the United Nations, grapevines are cultivated on almost 6.9 million hectares, producing 78 million tons of fruit. More than 70% of the crops are earmarked for wine production, while limited fractions are consumed as fresh (table grapes) or dried fruit (raisins), processed to grape juice, or distilled to brandy.

One of the major threats in viticulture is represented by the myriad of different pathogens and pests that may infect grapevine [1]. Among them, obligate intracellular parasites, such as viruses and phytoplasmas, are considered the most devasting infectious agents of grapevine. They drastically affect plant health and survival, causing heavy losses and significant reductions in yield and fruit quality, and often require costly approaches to mitigate damage [2]. 

Recently, the European Food Safety Authority (EFSA) Panel on Plant Health (PLH) established a comprehensive list of viruses and viroids of different plant species, including *Vitis vinifera* L. [3]. About 87 different viruses and viroids have been reported in grapevine to date [2,4]. Degenerative diseases (or decline), leafroll, and rugose wood, are among the most important viral diseases of grapevine, with major economic importance worldwide [5]. The degenerative disease known as fanleaf, including stunted, zig-zag shoot, and fan-shaped leaves, is mainly caused by European nepoviruses (e.g., grapevine fanleaf virus (GFLV)), whereas similar symptoms classified as “decline” are mainly associated with American nepoviruses [6]. Both clostero- and ampelovirus, which are described as grapevine leafroll-associated viruses (GLRaVs), are implicated in grapevine leafroll disease, although their etiological role has yet to be established [7,8]. Rugose wood is a complex of distinct diseases that have been associated with vitiviruses and foveaviruses [4,5].

Agricultural practices, mixed infections, spillover from reservoir host species, and impacts of a changing environment and global warming pose continuous challenges for the management of epidemics that result from the emergence of novel plant viruses [9]. In the last decade, the advent of high-throughput sequencing (HTS) technologies and their broad application in plant virology have prompted the discovery of a great number of newly emerging viruses and viral strains, which have been putatively associated with the onset of novel viral diseases [10]. Among these, grapevine Pinot gris virus (GPGV), which has been associated with grapevine leaf mottling and deformation disease (GLMD-d) [11], grapevine vein clearing virus (GVCV) [12], and grapevine red blotch virus (GRBV) [13], a quarantine pathogen actually included in the EPPO A1 list, are only a few of the recently reported grapevine viruses that are seriously threating viticulture and the wine industry [14]. 

## 2. Conventional Strategies for Controlling Viral Diseases in Grapevine

Grapevines infected by viruses cannot heal, since the plants are not able to defeat the virus(es). The outcome of the never-ending process of grapevine–virus co-evolution is extremely difficult to predict. A latent infection that has never caused severe symptoms or drastic physiological alterations may scale up quickly to a serious disease following environmental changes and/or in the presence of mixed infections [15]. Therefore, since no curative solutions and no resistant grapevine accessions are available, the management of viral diseases relies on preventive strategies. In this regard, the production of high-quality, virus-free planting material represents a crucial step to prevent the introduction of primary infections into vineyards [16]. Plantlets are usually produced through vegetative propagation of certified grapevine stocks, which have been subjected to appropriate sanitation processes that reduce (or eliminate) the presence of viruses. The sanitation procedures may involve thermal treatments, such as thermotherapy [17,18] and cryotherapy [19,20], or the administration of chemical compounds with antiviral activity through chemotherapy [21,22]. These methods are usually combined with in vitro cultures of meristems [23], shoot tips [24], and somatic embryos [25], which are generally virus-free due to the inability of viruses to infect the undifferentiated tissues [26]. 

Several factors may affect the outcome of sanitization procedures, significantly decreasing their efficacy. Thermotherapy is highly recommended against viruses located in parenchymatic tissues, while heat treatments are less effective for phloem-restricted viruses [26]. Similarly, the effectiveness of chemotherapy may greatly vary depending on the type and dosage of antiviral compound(s) used for the treatment, the grapevine cultivar being treated, and the susceptibility of the virus to specific drugs [22]. Moreover, explants treated with thermotherapy or chemotherapy may undergo thermal stress or show a significant accumulation of phytotoxic residues, which lead to a drastic decrease in the survival rate of plantlets [17]. Additionally, in vitro techniques show several disadvantages, among which the most important is the risk of genetic variations due to the use of high concentrations of plant growth regulators, repeated subculture, and shoot regeneration via callus formation [27,28]. Recently, with the availability of more effective diagnostic tools, such as HTS, it has been revealed that residual infections can persist for certain viruses even in plants regenerated from in vitro cultures, calling into question the efficacy of this technique in virus eradication [25,29]. 

Besides such prophylactic measures, agronomic practices, including rogueing of infected grapevines and replacement of entire diseased vineyards with healthy planting material [14] and the employment of beneficial microorganisms [30], provide alternative tools for the sustainable control of viral disease. Nevertheless, the high cost of the above-mentioned agronomic practices [31,32] and the scarce protection provided by the administration of biocontrol agents alone often require an integrated approach that involves the use of agrochemicals to control insect vectors [14]. 

**Figure 1:**
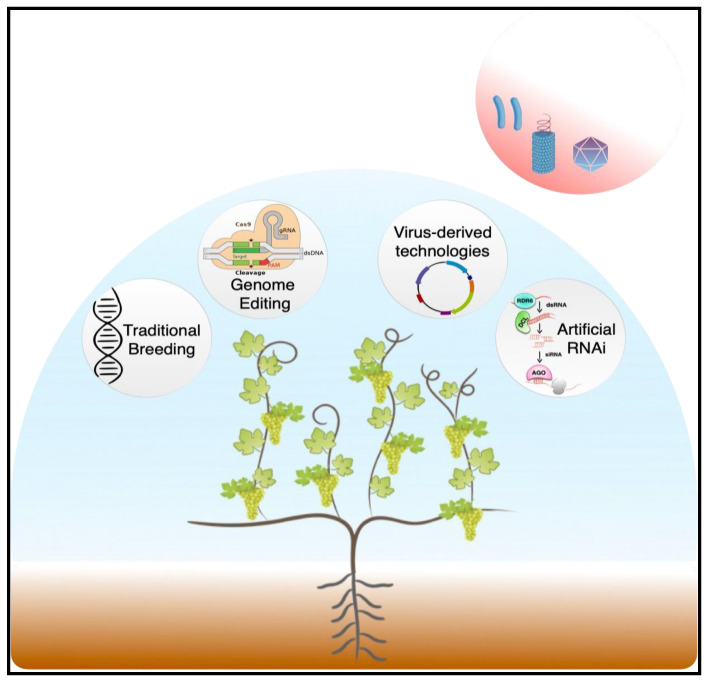
Graphical overview of the main biotechnological approaches currently available for the development of virus-resistant grapevines as sustainable alternatives for the management of endemic viral disease in vineyards.

According to the recently published European Green Deal plan, a 50% reduction in the use of agrochemical products is expected before 2030 [33]. Thus, the development of innovative disease-management strategies (Figure 1) that will satisfy the increasingly high demand for grapes while reducing chemical usage and maintaining production of high-quality fruit in an economically and environmentally sustainable way is the greatest challenge that viticulture must face in the coming years. 

## 3. Future Perspectives on Traditional Plant Breeding and Heritable Virus Resistance in Grapevine 

Traditional plant breeding has been widely employed to improve disease resistance and tolerance to abiotic stresses in elite cultivars of grapevine. This approach relies on the exploitation of the genetic variability among individuals of the same species by combining the desired traits into new and improved varieties. 

The Eurasian grapevine species *V. vinifera* is the most commonly used *Vitis* species in the grape and wine industry, favored for its superior aroma and flavor characteristics. Interestingly, *V. vinifera* has little or no genetic resistance against pathogens, rendering it highly susceptible to numerous diseases [34]. To date, no virus-resistant *Vitis* species have been identified, impeding the employment of traditional plant breeding to confer virus resistance in grapevine [16,35]. Nevertheless, the availability of sequenced genomes [36,37,38,39,40,41] and access to “*omic*” technologies [42] have significantly improved our knowledge of virus-resistance factors in grapevine. A recent discovery revealed that the Riesling clone 49 displays natural resistance to GFLV strain 13, although it is susceptible to the nematode vector *Xiphinema index* [43]. This resistance has been associated with a single recessive factor located on grapevine chromosome 1, which has been named *rgflv1*. The most important feature of the resistance provided by *rgflv1* is its monogenic determinism, which makes it stable through transmission to offspring and easy to use in breeding programs, despite being recessive. The discovery of *rgflv1* may pave the way for the first effective and environmentally friendly solution to control grapevine fanleaf disease through the development of new GFLV-resistant grapevine rootstocks, which was hitherto an unthinkable prospect [43], although further studies should be performed in order to assess whether the resistance provided by *rgflv1* against GFLV F13 strain may be effective against other GFLV strains.

Similarly, comparative genomics has revealed a myriad of viral sequences that are integrated, or ‘endogenized’, in the nuclear genomes of plants [44,45,46]. In 2005, Tanne and Sela revealed that a sequence homologous to the potato virus Y (PVY) coat protein (CP) gene was integrated and properly expressed in the genome of some grapevine varieties [47]. The genomic location/conformation of the PVY-CP-like sequence was reminiscent of a MITE-type retroelement because it was flanked by short direct repeats and was embedded in authentic grapevine sequences flanked by inverted repeats [47]. The authors speculated that recombined virus-derived sequences might have arisen from non-homologous recombination between a potyviral RNA and the RNA of a retrotransposable element that could potentially have occurred at some point in evolution [47]. Tanne and colleagues also hypothesized an alternative theory, suggesting that the potyviral sequences may have originated from a plant genome. However, this hypothesis was less convincing because potyviral sequences were found in only a few varieties; hence, they were unlikely to be native components of the grapevine genome [47]. 

The presence of numerous potential ORFs whose deduced protein sequences exhibited high identity to the protein sequences of distinct pararetroviruses (*Caulimoviridae* family) was identified through an in silico approach in annotated genomes of two Pinot noir plants [48]. The ORFs were scattered in an apparently random fashion among 11 of the 19 grapevine chromosomes, encoding for the reverse transcriptase (RT) and RNase H domains derived from unknown or extinct caulimoviruses and tungroviruses [48]. Since no pararetrovirus infections were found in grapevine, the authors suggested that the insertion of pararetroviral sequences conferred host resistance to these viruses, probably by triggering antiviral defense (i.e., RNA silencing) [48].

The molecular mechanism underlying heritable, homology-dependent virus resistance was first reported by Mette and colleagues [46]. According to their studies, over evolutionary time, several viral sequences have been integrated randomly by illegitimate recombination into host chromosomes. These endogenized viral sequences serve as templates to activate antiviral defense through homology-dependent gene silencing mechanisms, which allow the silencing of cognate viruses following infection. Silenced viruses can further trigger methylation and/or gene silencing of free viral genomes through the same homology-dependent processes, blocking virus replication and conferring long-term viral immunity to the host [46].

## 4. Early Activation of Antiviral Defense by Transgenic and Non-Transgenic Approaches

The early activation of antiviral defense in planta (i.e., RNA silencing) prior to the occurrence of viral infection represents an efficient strategy to protect plants from destructive viruses [49,50]. RNA silencing, together with RNA decay and RNA quality control, are the three major evolutionarily conserved RNA surveillance mechanisms, which target redundant/damaged/detrimental cellular and exogenous RNAs, regulating gene expression in eukaryotes [51]. In plants, these mechanisms also function as ancestral forms of intrinsic antiviral immunity [52]. In a broadly accepted antiviral RNA silencing model, plant-encoded Dicer/Dicer-like RNase-III-enzymes (DCLs) process viral double-stranded (ds)RNA into virus-derived small interfering RNA (vsiRNA) populations. vsiRNAs are loaded into host-encoded Argonaute (AGO) effector proteins as part of the RNA-inducted silencing complex (RISC) and guide it towards sequence-complementary viral RNA to execute antiviral silencing via endonucleolytic cleavage (i.e., post-transcriptional gene silencing (PTGS)) and/or translational repression (i.e., transcriptional gene silencing (TGS)) [50]. 

Historically, the early activation of antiviral RNA silencing before the occurrence of viral infection was achieved by the cross-protection strategy, which entailed plant pre-immunization with mild or avirulent viral strains that impeded the onset of severe viral infections due to the initiation of superinfection exclusion mechanisms [53,54]. Despite some successes, cross protection showed several drawbacks related to the use of avirulent strains, including negative impacts on fruit yield and quality, that made this strategy undesirable in the grape and wine industry [55].

Antiviral defense may also be activated transgenically through the host-induced gene silencing (HIGS) approach. This mechanism relies on the insertion of a viral transgene into the grapevine genome which readily activates RNA silencing [56]. Le Gall and co-authors were the first to induce resistance against the grape chrome mosaic virus (GCMV) by introducing the CP gene of the virus into 110 Richter rootstock [57]. Subsequently, transgenesis was exploited to produce grapevines resistant to different viruses, including Arabis mosaic virus (ArMV), grapevine viruses A and B (GVA and GVB), and GFLV [58,59,60,61]. 

Grapevine fanleaf disease threatens vineyards worldwide and it has always drawn a great deal of attention, which is likely due to the devasting impact and the challenging management of the disease in vineyards [6,62]. In the most severe cases, the disease may lead to yield losses of about 80%, with resulting economic losses estimated at USD 16,600 per hectare [63]. In 2010, Gambino and co-authors investigated the effects of GFLV CP expression in transgenic grapevine that was obtained using two distinct expression vectors: a pGA-CP+ plasmid carrying the full-length GFLV CP gene and a pGA-AS plasmid containing GFLV CP in the antisense orientation, resulting in an untranslatable form of the gene [59]. Bisulfite genomic sequencing analysis revealed significant levels of cytosine methylation in different loci of the viral transgene, which has been putatively associated with the activation of transcriptional silencing mechanisms by the plant against the transgene itself. Transgenic grapevines in which the transgene was silenced showed reduced virus resistance because virus spread after graft inoculation was not prevented [61]. Changes in DNA methylation following the introduction of foreign viral sequences have also been reported by Dal Bosco and co-authors, who revealed a negative correlation between viral resistance and levels of genome methylation, suggesting that the effectiveness of resistance conferred by transgenes of viral origin may be subject to epigenetic regulation [64]. These lines of evidence suggest that, besides the activation of antiviral RNA silencing against the cognate virus, a viral transgene may induce its own degradation, limiting or fully hijacking its protective function [65]. 

Technical and biological difficulties, the long time required, and strong rejection by consumers, as well as restriction by international legislation, have strongly hampered the wide exploitation of transgenic approaches in grapevine. 

Spray-induced gene silencing (SIGS) may be considered the “non-transgenic alternative” to the HIGS strategy [66]. This method relies on the exogenous application of artificial RNA molecules, such as hairpin-RNA (hpRNA) and/or dsRNA, onto plants to trigger gene silencing of target genes [66]. The SIGS approach has been already successfully employed in several plant species to induce resistance against different viruses [67,68]. With the exception of a few studies [69,70], there has been little exploitation of the SIGS approach in grapevine, and there have been no reports so far about its use as an antiviral strategy. Most likely, the scarcity of knowledge about the molecular mechanisms underlying the SIGS approach, specifically those regarding the delivery, functioning, and lifespan of exogenous RNA molecules in the plant, as well as the lack of suitable host targets, are still limiting this strategy. 

The triggering of RNA silencing via exogenous delivery of RNA molecules represents a more complex phenomenon, mostly due to the requirement for local and systemic spread of RNA molecules in plant tissues [71,72,73]. A major challenge is the improvement of the delivery and stabilization of dsRNA, especially for long-lasting crops cultivated in open fields, thus expanding the protective effect over a long time [74]. Moreover, the high costs linked to in vitro synthesis of dsRNA/hpRNA require the development of efficient and economically acceptable methods for large-scale production and purification of these molecules [75]. 

Elucidating the molecular mechanisms of SIGS and overcoming technical limits are key objectives for the successful future implementation of this novel biotechnological approach in agriculture. 

## 5. New Breeding Technologies (NBTs) to Induce Virus Resistance in Grapevine 

Since the early 2000s, new breeding technologies (NBTs) have evolved rapidly, providing a suite of innovative biotechnology-based methods designed to improve plant traits more rapidly and precisely than traditional plant breeding approaches [76,77]. 

Among NBTs, genome editing strategies are sparking increasing interest in agriculture, representing valuable tools for crop improvement and enhancement of disease resistance. These technologies entail the use of sequence-specific nucleases, such as zinc finger nucleases (ZFNs), transcription activator-like effector nucleases (TALENs), and the CRISPR/Cas9 system, which can be programmed to target virtually any DNA sequence of interest in the genome [78]. Compared with other genome editing tools, CRISPR/Cas is cost-effective, extremely precise, and reliable, offering robust and high-throughput genetic engineering.

The CRISPR system was first discovered in prokaryotes as a component of their adaptive immunity, conferring resistance to invading pathogens, such as bacteriophages and viruses [79]. 

The use of CRISPR/Cas9 as an RNA-programmable DNA editing platform requires single-guide RNA (sgRNA), consisting of a “*scaffold*” sequence, which directly binds the Cas9 nuclease, and a 20 nt sequence named “*spacer*”, which plays a crucial role in Cas9 recruitment [80]. Essential for cleavage is a three-nucleotide sequence motif immediately downstream at the 3′ end of the target region, known as the protospacer-adjacent motif (PAM; [80]). Once the Cas9-sgRNA complex binds to the target locus according to Watson–Crick base pairing, nuclease domains cleave DNA strands along with the PAM, introducing double-strand breaks (DSBs). Thereafter, a DSB may be repaired via the non-homologous end-joining (NHEJ; error-prone) DNA repair mechanism, which leads to the insertion/deletion of nucleotides causing gene knockouts [81,82], or via the homologous directed repair (HDR) mechanism, which, in the presence of a homologous DNA template, results in gene replacement knock-ins [83]. 

In recent years, the use of CRISPR/Cas9 to gain virus-resistant grapevine cultivars has raised significant interest. In this regard, Jiao and colleagues exploited this technology to target GLRaV 3, attempting to improve grapevine resistance to viral infection [84]. The authors selected ten target sites within the conserved ORFs of the GLRaV 3-Sau genome sequences, and five of these, namely, 5 kDa protein (p5), heat stimulated protein 70 homolog (Hsp70h), heat stimulated protein 90 homolog (Hsp90h), coat protein (CP), and minor coat protein (CPm), were used to synthesize sgRNAs for insertion into the chosen vectors [84]. Despite results indicating that all the tested constructs were able to attenuate GLRaV 3 infection, vectors harboring GLRaV 3 CP and Hsp70h proteins exhibited the most robust inhibition efficiencies and were able to be further engineered to generate GLRaV 3-resistant grapevines [84]. The study confirmed that CRISPR/Cas technology could be successfully exploited to attenuate viral infections in grapevine, providing new avenues to control GLRaV 3 or other RNA viruses in woody crops. 

Instead of targeting viral RNA (virus-mediated resistance), CRISPR/Cas technology may also be employed to manipulate the plant genome (plant-mediated resistance) by editing gene sequences that are putatively involved in viral infections [85]. Among these, translation initiation factors play important roles in most steps of viral infection, participating in the synthesis of viral proteins which cannot be encoded by viruses themselves due to their limited coding capacity, regulating viral replication, and facilitating local and systemic movement of viruses (for a review, see [86]). Although no practical application of CRISPR/Cas9-mediated knockouts of translational factors involved in viral infections has been reported for grapevine, the great number of studies that attest its efficiency [87,88,89,90] are laying the foundations for the use of this technology in the sustainable control of viruses.

Nevertheless, CRISPR/Cas9 technology also shows several limitations that should be considered in order to establish the most effective antiviral strategies. The occurrence of off targets represents the main drawback of the CRISPR/Cas system because they may lead to the knockout of essential host factors, resulting in the impairment of plant development, or, in the most severe cases, be lethal to the plant [91]. 

Despite the frequency of off-target mutations in grapevine due to the CRISPR/Cas system, they are likely insignificant compared to variations caused by tissue culturing and/or *Agrobacterium*-mediated transformation, and they may be further reduced by the use of Cas variants with higher specificity [92] or by employing catalytically inactive Cas [93,94]. 

Moreover, to avoid the evolution of viruses with mutations allowing escape from CRISPR/Cas cleavage, an accurate selection of reliable sgRNA sites is strongly recommended [95,96]. In this regard, multi-locus gene editing and/or editing of non-coding loci in viral genomes has been found to significantly reduce mutation rates, minimizing the evolution of new viral strains [96].

## 6. Grapevine Viruses—From Targets to Tools: The Employment of Viral Vectors in Emerging Biotechnologies

Thanks to their small-sized genomes and their autonomous replication, plant viruses are ideal for engineering as vectors for the expression of heterologous proteins, avoiding stable transformation and the production of genetically modified (GM) plants [97,98]. Several RNA viruses infecting grapevine have been successfully engineered and repurposed as viral vectors. Among these, the most important are grapevine virus A (GVA), GFLV, ArMV, and grapevine leafroll-associated virus 2 (GLRaV 2).

Infectious cDNA clones of GLRaV-2 have been exploited in a reverse genetic study to investigate the functional role of papain-like L1 and L2 proteases in the viral replicative cycle, assessing their eventual involvement in the systemic spread of the virus in *N. benthamiana* plants [99]. The entire monopartite virus genome was retrotranscribed and inserted into a binary vector for *Agrobacterium tumefaciens*-mediated expression between the cauliflower mosaic virus (CaMV) 35S promoter and ribozyme sequence immediately upstream of the nopaline synthase terminator sequence [99]. The resulting full-length GLRaV-2 clone was further modified to express the GFP reporter gene whose open reading frame (ORF) was placed immediately upstream of the ORF encoding the coat protein (CP). With the aim of restoring GLRaV-2 expression, the beet yellows virus (BYV) subgenomic (sg) RNA promoter of the CP gene was placed immediately downstream from the GFP ORF [99]. 

In RNA virus-based vectors, the insertion of sg promoters belonging to closely related species has been shown to be preferable to promoter duplication because of the rapid loss of identical sequences due to homologous recombination [100]. In addition to the infectious clone, GLRaV 2 replicons were produced by deletion of a region of the viral genome that was doubly labeled with GFP and GUS reporters [99]. As expected, the full-length cDNA clone was able to systemically infect the phloem tissues of *N. benthamiana*. Elsewhere, the GLRaV 2 replicons did not show tropism, since the expression of reporter genes was exclusively detected in proximity to the agroinfiltrated leaf areas [99]. 

This first generation of full-length cDNA clones of GLRaV 2, however, was not able to systemically infect grapevine and therefore could not be exploited as a viral vector in this host [99]. The maintenance of GLRaV 2 to produce viral RNA for the synthesis of full cDNA by serial infections of *N. benthamiana* led to the adaptation of the virus to the new host plant, selecting a viral variant of GLRaV-2 that was unable to infect grapevine [101]. 

A new GLRaV 2 vector was generated from RNA collected from Pinot noir plants [102]. This novel version, named vLR2-GFP, was able to systematically infect *V. vinifera* plants. Expression of GFP was monitored in phloem cells of roots and leaves, as well as in berries. In contrast to other viral vectors based on tobamoviruses and potyviruses, vLR2-GFP revealed considerable genetic stability, persisting in grapevine and maintaining the exogenous insert for more than a year after agroinfiltration [102]. Furthermore, the vLR2-based vectors can be effectively transmitted to different grapevine varieties that are recalcitrant to agroinoculation by grafting [102]. 

This second generation of GLRaV 2 vectors was successfully used to promote VIGS. Instead of (or immediately after) the GFP expression cassette, the introduction of cDNA sequences encoding the phytoene desaturase (PDS) or the subunit I of magnesium-protoporphyrin chelatase (ChlI) promoted suppression of the cognate gene in grapevine through the activation of gene silencing mechanisms. The simultaneous expression of GFP and the adjacent coding sequence targeting the grapevine’s endogenous genes allowed observation of the VIGS response in relation to viral tropism in the plant. After infection, chlorophyll bleaching was observed first in the veins where GLRaV 2 replicated, then, following transitivity, in phloem tissues far from the viral replication site. VIGS persisted for months after viral inoculation, with symptoms appearing cyclically and depending on the growth stage of the plant, tissue differentiation, and viral spread [102]. 

Moreover, since GLRaV 2 has no natural vectors and can only be transmitted by grafting, any potential biosafety risk(s) related to the release and fate of GLRaV 2-derived vector(s) in the environment is limited. 

GVA, belonging to the genus *Vitivirus,* can also be used as a viral vector to express exogenous genes or for VIGS experiments [103]. The GVA-derived vector was initially developed in *N. benthamiana* to express reporter genes (i.e., GUS) or proteins of unrelated viruses [104]. As a preliminary approach, the complete cDNA sequence of GVA was inserted into a plasmid carrying the T7 phage promoter for in vitro transcription. Then, its 3′ terminal portion comprising the ORFs -MP, -CP, and the -3′ untranslated region (UTR) was substituted with that of another strain of GVA, with the addition of its MP sg promoter. The resulting chimeric virus thus possesses two different MP sg promoters separated by a spacer carrying a restriction site sequence for the insertion of exogenous codifying sequences [104]. 

In subsequent work, the vector was further engineered to perform VIGS experiments in *N. benthamiana* and in grapevine plants. The viral cDNA was inserted into a binary vector under the transcriptional control of two CaMV-35S promoters and a single CaMV-35S terminator. To assess the effectiveness of this VIGS approach, a sequence homologous to partial regions of PDS genes from either *N. benthamiana* or grapevine was introduced in place of the reporter gene cassette [103]. In *N. benthamiana* plants agroinfiltrated with a GVA-derived vector carrying the PDS construct, chlorophyll photo-bleaching was limited to the leaf veins [103]. Surprisingly, in grapevines agrodrenched with the same construct [105], symptoms were first observed at the leaf margins and then all over the leaf blades [103].

The foveavirus grapevine rupestris stem pitting-associated virus (GRSPaV), which, like GVA, belongs to the *Flexiviridae* family, has also been engineered to express enhanced-GFP (eGFP) in both *N. benthamiana* and grapevine [106]. The GRSPaV-derived vector included the CaMV-35S promoter and the Nos terminator to control its transcriptional activity. In addition, the Hepatitis D virus ribozyme sequence was placed before the Nos terminator to promote transcribed RNA cleavage and remove the poly-A tail. The eGFP sequence was introduced into the viral vector after the ORF encoding the triple gene block, under the transcriptional control of the CP sgRNA promoter of a different GRSPaV strain. Whereas filamentous virions were observed in *N. benthamiana* in proximity to the agroinfiltrated area, in grapevine the GRSPaV viral vector harboring the eGFP construct was able to spread systemically, since fluorescence signals were readily reported in distal tissues as well. However, confocal microscopy observations showed eGFP fluorescence only in restricted clusters of a few cells. This aspect has been putatively correlated either with a low titer of virus-derived vector or with loss of the eGFP expression cassette. Despite its potential, the ability of the GRSPaV viral vector to induce host gene silencing has still not been analyzed [106].

Additionally, the tombusvirus grapevine Algerian latent virus (GALV) has been employed as a viral vector. GALV was initially detected in grapevine [107] and later found in statice (*Limonium sinuatum*) and nipple fruit (*Solanum mammosum*) [108,109]. GALV is a water-borne virus and can be found free in the environment, persisting for long periods outside the host [110]. Infectious clones of GALV were assessed initially in *N. benthamiana* and several grapevine varieties, including Syrah, Brachetto, Nebbiolo, Sultana, and Corvina, which were found to be susceptible to systemic infection by the synthetic virus [111]. Afterward, the infectious clone was engineered into a viral vector by replacing the original CP gene from the viral genome with an exogenous protein expression cassette [112]. In order to assess the ability of the novel GALV vector to infect *N. benthamiana* plants, the GFP sequence was added to the construct, which was named pGMG [112]. The necrotic phenotype of the infected cells, which has been attributed to the presence of the symptom-determining p19 viral suppressor, seemingly blurred any GFP signal [112]. A weak and short-lived GFP signal was detected in infiltrated leaves of *N. benthamiana* following knockout of the p19 suppressor [112]. This novel version of the GALV vector, named pGMG19m, was used to induce silencing of the ChlI gene in *N. benthamiana* plants. The typical chlorophyll bleaching phenotype was clearly detected in systemic leaves of infiltrated plants, demonstrating the potential of the GALV-derived pGMG19m vector to be employed in a VIGS approach [112]. Despite the promising results achieved in the experimental host, the VIGS activity of the GALV-derived pGMG and pGMG19m vectors in *V. vinifera* has yet to be assessed.

DNA viruses can also be employed as vectors for the delivery of genetic information into host cells and/or for gene editing technology [113]. The *Geminiviridae* family represents the largest family of DNA viruses, consisting of numerous viral species characterized by circular ssDNA genomes [114]. Geminivirus-based vectors, also known as non-infectious geminiviral replicons (GVRs), are self-replicating, cell-autonomous DNA vectors obtained by genetic manipulation of infectious clones [113,115]. In the context of the previously described NBTs, the use of GVRs offers key advantages for precise editing via homology-directed repair (HDR; [113]). When used to deliver site-specific nucleases and donor templates, GVRs noticeably increase the frequency of HDR in host cells [113]. Besides the higher expression of site-specific nucleases and the greater concentration of donor templates afforded by the GVRs, the occurrence of pleiotropic effects related to viral replication proteins renders the cell environment more favorable to HDR [113]. 

Although some geminirus-derived vectors, such as tomato yellow leaf curl virus (TYLCV) and bean yellow dwarf virus (BeYDV), have already been successfully employed in grapevine studies involving functional genetic [116] and genome editing assays [117], no viral vector has been developed so far from geminiviruses that naturally infect grapevine. Nevertheless, these viruses may provide great advantages in the context of modern biotechnologies. The small size of their DNA genome, as well as its conserved organization, and their wide host range are just a few of the valuable features that this viral genus has to offer [118]. DNA vectors, such as those derived from geminiviruses, have proven to be more stable than RNA virus-derived vectors, allowing them to be directly inoculated without the use of *Agrobacterium* as a carrier [118]. 

## 7. Concluding Remarks

Environmental change, global warming, and trade in propagation material are only a few of the factors that are favoring the onset and spread of novel viruses [119,120,121], which are seriously threatening viticulture worldwide. The containment of viral diseases in vineyards represents a difficult challenge, and huge efforts have been invested in the development of sustainable control strategies. So far, prophylactic measures that entail the planting of certified virus-free grapevines and the use of agrochemicals to prevent the spread of insect vectors are still the only effective tools against grapevine viruses. 

Given the impending drastic reduction in the use of chemical pesticides (at least 50% by 2030, according to the Green Deal objectives of EU policy), implementation of the innovative biotechnological tools that have been developed so far represents the only promising strategy for the sustainable control of disease in vineyards.

The scarcity of sources of viral resistance in the *Vitis* germplasm, severe inbreeding depression, long lifecycle, and lack of knowledge of resistance genes or the genetic architectures of the relevant traits have strongly limited the use of traditional plant breeding in controlling grapevine viruses, thus promoting the use of transgenic strategies [35].

In grapevine, transgenic approaches are highly laborious due to its recalcitrance to both transformation and in vitro regeneration. Moreover, these techniques are expensive and time-consuming, requiring several years, to produce new varieties. Besides these technical and biological limitations, the constitutive expression of viral transgenes with the potential to induce virus resistance was also found to drastically interfere with plant development, causing serious morphological and physiological anomalies in transgenic grapevine [122]. These aspects, along with current EU regulations that still restrict transgenesis in many countries, strongly limit the use of these approach in agriculture. 

The powerful new breeding technologies (NBTs) are expected to make substantial contributions to tackling the future challenges faced by the grapevine and wine industry. Among them, CRISPR/Cas9-mediated genome editing represents a promising technology for the development of virus-resistant varieties. However, three main drawbacks are impeding the implementation of genome editing: controversial regulation, high costs, and social and market acceptance. The regulatory aspects have a major influence on the others, since the lack of a clear recognition of NBT-derived varieties as either genetically modified (GM) or non-GM organisms may encourage their acceptance, promoting investments in both research and commercialization. In fact, unlike classical transgenic approaches, NBTs include a range of new strategies, such as genome editing technologies, such that the resulting varieties may not be considered genetically modified organisms (GMOs), depending on how the NBTs are applied. 

As an alternative to NBTs, plant immunization via the exogenous application of RNA molecules that are capable of activating plant defenses prior to the establishment of pathogenic infections (i.e., the SIGS approach) provides a novel, non-transgenic means for the sustainable control of disease in grapevine, which may replace the use of agrochemicals in vineyards.
